# Supplementary material for: Phylogenetic analysis of two single-copy nuclear genes revealed origin of tetraploid barley Hordeum marinum
Source: PLoS One. 2020 Jun 30;15(6):e0235475. doi: 10.1371/journal.pone.0235475 (PMC7326175; doi:10.1371/journal.pone.0235475)
Supplement: S1 Table — (DOC) [file pone.0235475.s001.doc]

**S1 Table．Taxa from *Aegilops*, *Psathyrostachys*, *Secale*, *Taeniatherum*, *Australopyrum*, *Triticum*, *Pseudoroegneria* and *Hordeum* used in this study.**

| Species | Accession no. | Genome | Origin | *TRX* | *WAXY1* |
| --- | --- | --- | --- | --- | --- |
| Aegilops speltoides | PI487232 | S | The Middle East | ﹣ | JX679014 |
| Australopyrum retrofractum |  | W | New South Wales,Australia | ﹣ | AF079272 |
| H.bulbosum | IB5-14 | H | Morocco | AB509232 | ﹣ |
|  | 710-17 | H | Morocco | AB509231 | ﹣ |
| H.vulgare subsp.spontaneum | OUH620 | H | Mexico | AB509229 | ﹣ |
| H.vulgare subsp.vulgare | Chikurin Ibaraki1 | H | Japan | AB509228 | ﹣ |
|  | Bonus | H | Sweden | AB509227 | ﹣ |
| H.vulgare subsp.spontaneum | OUH643 | H | Mexico | ﹣ | AB118808 |
| H.vulgare subsp.vulgare | TR306 | H | Unknown | ﹣ | KC963118 |
| H.vulgare var.distichon | Z143 | H | Qinghai,Tibet | ﹣ | KT356854 |
| H.bulbosum | H3878 | H | Italy | ﹣ | HQ619466 |
|  |  | H | North American | ﹣ | AY010962 |
| H.chilense |  | I | Chile | ﹣ | EU282318 |
| H.brevisubulatum |  | I | North American | ﹣ | AY010961 |
| H.violaceum |  | I | North American | ﹣ | AY010964 |
| H.pusillum |  | I | USA | ﹣ | EU282321 |
| H.patagonicum subsp.setifolium | H1357 | I | Argentina | ﹣ | HQ619460 |
| H.patagonicum subsp.patagonicum | H1319 | I | Argentina | ﹣ | HQ619459 |
| H.patagonicum subsp.magellanicum | H6209 | I | Argentina | ﹣ | HQ619468 |
| H.patagonicum subsp.mustersii | H1358 | I | Argentina | ﹣ | HQ619461 |
| H.patagonicum subsp.santacrucense | H1493 | I | Argentina | ﹣ | HQ619462 |
| H.comosum | H1181 | I | Argentina | ﹣ | HQ619456 |
| H.erectifolium | H1150 | I | Argentina | ﹣ | HQ619455 |
| H.cordobense | H6429 | I | Argentina | ﹣ | HQ619469 |
| H.flexuosum | H1133 | I | Argentina | ﹣ | HQ619454 |
| H.muticum | H958 | I | Bolivia | ﹣ | HQ619453 |
| H.intercedens | H1940 | I | USA | ﹣ | HQ619463 |
| H.brevisubulatum subsp.violaceum | H315 | I | Iran | ﹣ | HQ619450 |
| H.euclaston | H1263 | I | Argentina | ﹣ | HQ619457 |
| H.brachyantherum subsp.californicum | H1954 | I | USA | AB509252 | ﹣ |
|  | H3317 | I | USA | AB509251 | ﹣ |
| H.pubiflorum | H1296 | I | Argentina | AB509254 | ﹣ |
| H.chilense | Camb.line1 | I | Unknown | AB509253 | ﹣ |
| H.roshevitzii | H9152 | I | China | AB509250 | ﹣ |
| H.bogdanii | H4014 | I | Pakistan | AB509249 | AB154358 |
| H.pusillum | H2038 | I | New Mexico, USA | AB509255 | ﹣ |
| H.brevisubulatum subsp.violaceum | H316 | I | Iran | AB509248 | ﹣ |
|  | H304 | I | Turkey | AB509247 | ﹣ |
|  | H801 | Xu | Iran | ﹣ | HQ619452 |
| H.murinum subsp.glaucum | JIC line71 | Xu | Unknown | AB509241 | ﹣ |
|  | H10289 | Xu | Tajikistan | AB509242 | ﹣ |
| H.murinum subsp.murinum | H614 | XuXu | Greece | AB509243 | ﹣ |
|  |  | XuXu | Greece | AB509245 | ﹣ |
| H.murinum subsp.leporinum | H509 | XuXu | Spain | AB509246 | ﹣ |
| H.marinum subsp.gussoneanum | H819 | XaXa | Turkey | AB509237 | ﹣ |
|  |  | XaXa | Turkey | AB509239 | ﹣ |
|  | H2303 | XaXa | USA | AB509238 | ﹣ |
|  |  | XaXa | USA | AB509240 | ﹣ |
| Psathyrostachys juncea | H10108 | Ns | Russia | AB509256 | ﹣ |
| Psathyrostachys juncea | PI206684 | Ns | Europe | ﹣ | AF079280 |
| Pseudoroegneria spicata | PI232117 | St | U.S.A. | ﹣ | AF079281 |
| Secale cereale | RY12 | R | Turkey | ﹣ | KC572695 |
| Taeniatherum caput-medusae | Taecap2b | Ta | Russia | ﹣ | AY360847 |
| Triticum urartu |  | A | Mediterranean | ﹣ | KF612975 |

**Note:** The *TRX* and *WAXY1* sequences from polyploid species of *Hordeum* were sequenced and analyzed.

*The plus sign (+) indicates that sequence data has been recovered; the minus sign (−) indicates no sequence data has been recovered.
