# Supplementary material for: Phylogenetic analysis of two single-copy nuclear genes revealed origin of tetraploid barley Hordeum marinum
Source: PLoS One. 2020 Jun 30;15(6):e0235475. doi: 10.1371/journal.pone.0235475 (PMC7326175; doi:10.1371/journal.pone.0235475)
Supplement: S2 Table — (DOC) [file pone.0235475.s002.doc]

**S2 Table.** Maximum Likelihood fits of nucleotide substitution models.

| **Model** | **Parameter** | **BIC** | **AICc** | **lnL** | **G** | **R** | **F-A** | **F-T** | **F-C** | **F-G** |
| --- | --- | --- | --- | --- | --- | --- | --- | --- | --- | --- |
| **HKY+G** | 140 | 10104.546 | 8856.6653 | -4287.9741 | 2.2754275 | 1.2941525 | 0.2140023 | 0.287257 | 0.2625247 | 0.236216 |
| **TrN+G+I** | 156 | 4398.8743 | 3136.9560 | -1411.4676 | 0.8108074 | 1.6819330 | 0.2020003 | 0.2178636 | 0.2698803 | 0.3102558 |

Note: HKY (Hasegawa-Kishino-Yano), TrN (Tamura-Nei), BIC (Bayesian Information Criterion), AICc (Akaike Information Criterion, corrected), lnL (Maximum Likelihood value), G (Gamma distribution), R (Assumed or estimated values of transition/transversion bias), F (frequence).
