# Supplementary material for: Phylogenetic analysis of two single-copy nuclear genes revealed origin of tetraploid barley Hordeum marinum
Source: PLoS One. 2020 Jun 30;15(6):e0235475. doi: 10.1371/journal.pone.0235475 (PMC7326175; doi:10.1371/journal.pone.0235475)
Supplement: Attachment — Submitted filename: Response to reviewers.doc [file pone.0235475.s003.doc]

June 15, 2020

Dear Wujun Ma:

Thanks for providing us with an opportunity to revise our manuscript. We have taken all comments from reviewers into consideration, and made a revised version of this manuscript. We invited Professor Genlou Sun (Saint Mary University of Canada) and Professor Hong Luo (Clemson University) to revise this manuscript. I am sending you our revised manuscript along with a point-by-point description of the changes made for your reviewing. I hope that our manuscript will meet your standards for publication in Plos one.

Sincerely yours,

Xifeng Ren

**Description of the changes**

Journal Requirements:

When submitting your revision, we need you to address these additional requirements.

1. Please ensure that your manuscript meets PLOS ONE's style requirements, including those for file naming. The PLOS ONE style templates can be found at

<https://journals.plos.org/plosone/s/file?id=wjVg/PLOSOne_formatting_sample_main_body.pdf> and <https://journals.plos.org/plosone/s/file?id=ba62/PLOSOne_formatting_sample_title_authors_affiliations.pdf>

**Response:** Thanks for your suggestion. Changes were made according to the suggestions.

2. We suggest you thoroughly copyedit your manuscript for language usage, spelling, and grammar. If you do not know anyone who can help you do this, you may wish to consider employing a professional scientific editing service.  

Whilst you may use any professional scientific editing service of your choice, PLOS has partnered with both American Journal Experts (AJE) and Editage to provide discounted services to PLOS authors. Both organizations have experience helping authors meet PLOS guidelines and can provide language editing, translation, manuscript formatting, and figure formatting to ensure your manuscript meets our submission guidelines. To take advantage of our partnership with AJE, visit the AJE website ([http://learn.aje.com/plos/](about:blank)) for a 15% discount off AJE services. To take advantage of our partnership with Editage, visit the Editage website (www.editage.com) and enter referral code PLOSEDIT for a 15% discount off Editage services.  If the PLOS editorial team finds any language issues in text that either AJE or Editage has edited, the service provider will re-edit the text for free.

Upon resubmission, please provide the following:

a) The name of the colleague or the details of the professional service that edited your manuscript

b) A copy of your manuscript showing your changes by either highlighting them or using track changes (uploaded as a *supporting information* file)

c) A clean copy of the edited manuscript (uploaded as the new *manuscript* file)

**Response:** Thanks for your suggestion. Changes were made according to the suggestions. We have carefully edited this manuscript.

**Reviewer #1**

Reviewer #1: The study used two single-copy nuclear gene markers to classify a collection of 33 Hordeum marinum species, in order to draw a phylogenetic consensus map and reveal the origin of the tetraploid subspecies in Hordeum marinum. If my synopsis of the article is wrong, please discard my comments and find an alternative reviewer.

It is a potentially article because of the novelty of the work and new findings revealed by one of the gene marker derived from the TRX gene. Compared to previous work [refer to reference number 3], the study has found similar results that the gussoneanum (4x) was clustered into two clades by the TRX gene marker, one of the clade was grouped with the Xa genome, but the other clade was not grouped into any genome. In comparison, by using the WAXY1 gene marker, all of the gussoneanum (4x) were grouped together, but overlapped with some accessions from the gussoneanum (2x) group. My question is: is that possible the unclustered clade may carry a new genome that was never reported before, or the overlapped group the gussoneanum (4x) and gussoneanum (2x) accessions is the transition stage from the gussoneanum (2x) type evolving to the gussoneanum (4x) type?

By reading the manuscript, the answer seems unclear.
**Response:** Thanks for your suggestion. Because the limited number of genes used in the experiment, the origin of tetraploids of *H. marinum* subsp*. gussoneanum* has not been fully explored. Yes, the unclustered clade may carry a new genome that was never reported before. It may come from an extinct species. Similar results have appeared in previous literature reports.

Comments on other parts of the manuscript:
1. It is unclear why nuclear TRX and WAXY1 gene markers were selected out of numerous other genes. Authors must have very sound reasons to do so. As a result, the reasons should be elaborated in the introduction part. Why they are selected? If they have been used for classification before, what are the main findings? If they were not used before, what's the advantages of using them and expected outcomes?
**Response:** Thanks for your comments. We have added this information in the introduction. The nuclear *TRX* and *WAXY1* genes were selected as markers because they are relatively conservative during the evolution of *Triticeae*, and they have also been used in phylogenetic studies.

2. There are a few places where the font of the words are different from the rest. I suggest using line numbers on pages to identify sentences when resubmit.
**Response:** Thanks for your suggestion. Changes were made according to the suggestions.

3. Reference 25: format
**Response:** Thanks for your suggestion. We have corrected the format error of the reference.

4. Fig.1 and Fig.2 sentence "Geographic distribution of ...." sentence is incomplete, the denotation for the "red line" is missing.
**Response:** Red is only labeled as tetraploids of *H. marinum* subsp*. gussoneanum*, and its geographical distribution is shown in Table 1. For readability, we have changed the red label to black, with pictures and notes.

5. The last paragraph or "conclusion" paragraph needs to rewrite. This part should only have results from the study and concluding remarks from the authors, so the citation is redundant. Again, the word "angiosperms"
is inappropriate in presenting here.
**Response:** Thanks for your suggestion. We have revised the conclusion.

**Reviewer #2**

Reviewer #2: This paper was written very well. Sea barley Hordeum marinum is an important germplasm resource. This paper suggested that tetraploid more likely originated from the diploids of H. marinum subsp. gussoneanum and the other ancestor that might be an extinct unknown diploid species. Homogenization of gene in tetraploids also occurred after polyploidization as both TRX and WAXY1 sequences in some accessions of tetraploid H. marinum subsp. gussoneanum cannot be distinguished, indicating the complicated evolution of this tetraploid. Line H824 was an interesting line, the gene was grouped into one cluster with 2X for both genes. But other 4X lines changed when they were analysed with two distinct genes. It means that the more genes used in the study, the better results will be obtained. Discuss this in the paper. **Response:** Thanks for your suggestion. We added relevant discussion on this.

There are some other minor issues which need to be addressed.
Page 8:
Abstract: "H. marinum subsp. gussoneanum, diploid marinum" from two sentences, insert “and”.
**Response:** Thanks for your suggestion. Changes were made according to the suggestions.

P13: Rewrite this sentence “To reveal the genetic differentiation between the diploid H. marinum subsp. Gussoneanum and the diploid H. marinum subsp. marinum used TRX, genetic analysis and neutrality test in three populations were performed.”
**Response:** Thanks for your suggestion. Changes were made according to the suggestions.

P13: change 1 from “While just 1 haplotype” to one. 1 to 9 should be written out.
**Response:** Thanks for your suggestion. Changes were made according to the suggestions.

Reference:
Italic Hordeum marinum for the reference 4. Double check other references.

**Response:** Thanks for your suggestion. We have corrected the format error of all reference.
